# Molecular dynamics study of the inhibitory effects of ChEMBL474807 on the enzymes GSK-3β and CDK-2

**DOI:** 10.1007/s00894-015-2627-z

**Published:** 2015-03-10

**Authors:** Przemysław Czeleń, Beata Szefler

**Affiliations:** Department of Physical Chemistry, Collegium Medicum, Nicolaus Copernicus University, Kurpinskiego 5, 85-950 Bydgoszcz, Poland

**Keywords:** ATPase, Competitive inhibition, Similarity, Molecular dynamics, CDK-2, GSK-3β

## Abstract

Indirubin derivatives and analogs comprise a significant group of ATP-competitive inhibitors. The inhibitory effects of ChEMBL474807 (1-(4-amino-1,2,5-oxadiazol-3-yl)-5-(piperidin-1-ylmethyl)-*N*′-(pyridin-4-ylmethylene)-1*H*-1,2,3-triazole-4-carbohydrazide) on two enzymes, namely glycogen synthase kinase-3β (GSK-3β) and cyclin-dependent kinase-2 (CDK-2), were analyzed. The close resemblance of the amino acid sequences of these two enzymes (with 25 % identity and 41 % similarity) explains why indirubin derivatives are inhibitors of both of the enzymes studied. The docking and molecular dynamics investigation performed here led to the identification of the interactions responsible for stabilizing the ligand ChEMBL474807 at the active sites of the enzymes considered. The structural and energetic data collected during our investigations clearly indicate that there are important differences in the behavior of the ligand at the two active sites investigated here.

## Introduction

Indirubin is a component of *Indigo naturalis*, namely the red-colored 3,2′-bisindole isomer. It is an active ingredient used in a traditional Chinese medicine for the treatment of chronic diseases. Small bioactive molecules that inhibit cellular replication machanisms are currently being exploited for novel therapeutics. Indirubin derivatives such as indirubin-3′-oxime have been investigated in this respect. Multiple effects of indirubin-3′-oxime on the activity of mitochondrial ATPase have been observed, indicating that this small molecule interferes with cell function [1]. Studies performed in vitro and on animals have shown that indirubin and its derivatives possess anti-inflammatory [2], antitumor [3, 4], antiangiogenic [5], and neuroprotective [6] effects. They also inhibit cyclin-dependent kinases in tumor cells [7, 8], enhance the cytotoxic effects of doxorubicin [9], and represent an efficient treatment for psoriasis [10]. In a study of patients with head and neck cancer, indirubins were found to reduce the mucosal damage caused by radiation therapy [11, 12]. Indirubins inhibit DNA synthesis during cell proliferation in the late-G1 and G2/M phases by selectively inhibiting cyclin-dependent kinases (CDK) [12, 13] by competing at the ATPase-binding site [8]. Indirubin inhibits the assembly of microtubules involved in cell reproduction [14]. Its anti-inflammatory effects seem to originate from its inhibition of interferon gamma [2]. Indirubin is a constituent of *Indigofera tinctoria*; the synthetic form of indirubin was shown to have a similar effectiveness against CML to that of natural indirubin [15–17]. Indirubin also inhibits the growth of the human promyelocytic leukemia cell line HL-60 [18] and human HT-29 colorectal cancer cells [19], and it modulates the proliferation of keratinocytes in patients with psoriasis [20]. Mitochondrial dysfunction due to indirubin-3′-oxime may be an important mechanism for cell cycle arrest in human neuroblastoma cells [21].

**Fig. 1a–b:**
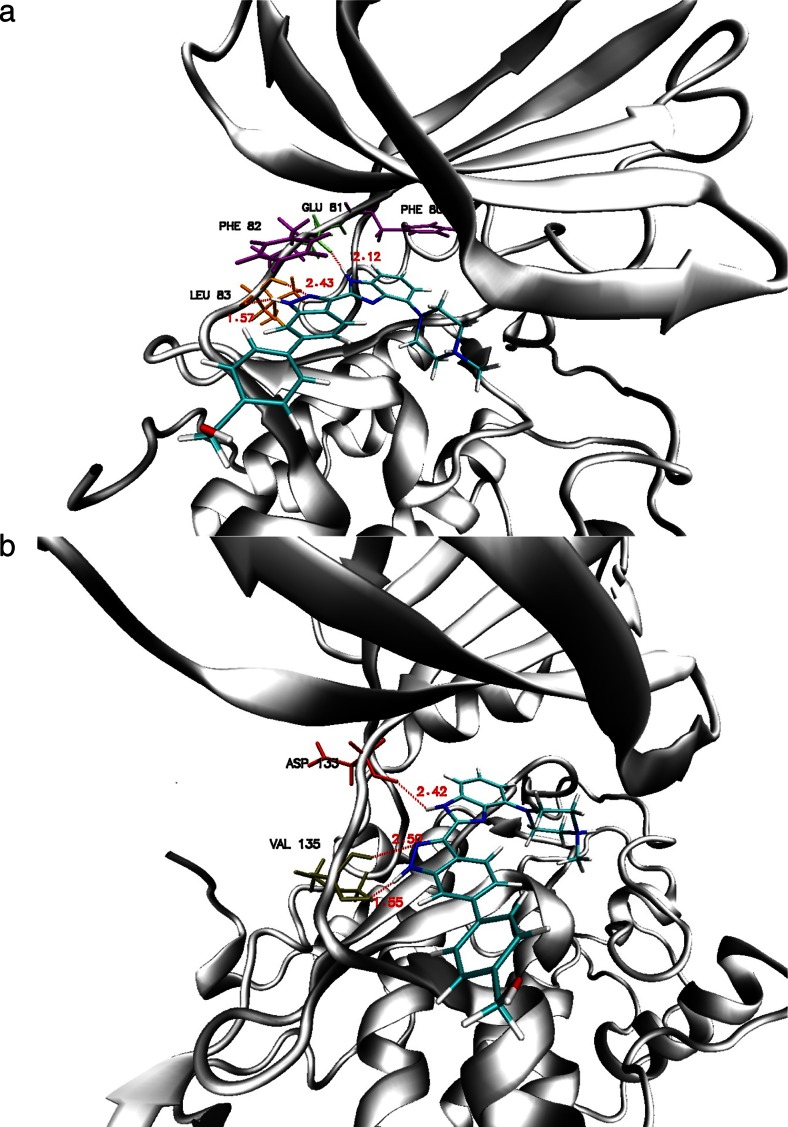
The most important interactions observed between ChEMBL474807 and the active sites in the enzymes CDK-2 (**a**) and GSK-3β (**b**)

During the investigations reported in the present paper, the inhibitory effects of ChEMBL474807 (1-(4-amino-1,2,5-oxadiazol-3-yl)-5-(piperidin-1-ylmethyl)-*N*′-(pyridin-4-ylmethylene)-1*H*-1,2,3-triazole-4-carbohydrazide) on two enzymes, namely glycogen synthase kinase-3β (GSK-3β) and cyclin-dependent kinase-2 (CDK-2), were analyzed (see Fig. 1). The close resemblance of the amino acid sequences of these two enzymes (with 25 % identity and 41 % similarity) [22] proved that indirubin derivatives are inhibitors of both enzymes [23]. This situation necessitates a search for selective kinase-inhibiting compounds [24].

## Methods

During the docking simulations, the crystal structures of CDK-2 (PDB ID: 1E9H) [25] and GSK-3β (PDB ID: 1Q41) were used [26], both of which were downloaded from the Brookhaven Protein Database (PDB). Docking was realized using a united-atom scoring function implemented in AutoDock Vina [27]. Before the docking stage, all of the water molecules were removed from the crystal structures of the considered enzymes, as were all of the nonpolar hydrogen atoms. All preparation steps were performed using the AutoDock Tools software package. After determining the grid box dimensions for each of the active sites considered, the docking procedure was repeated ten times. The conformations of the ligand ChEMBL474807 at the active sites of the enzymes (obtained during the docking stage) were almost identical, and reproduced the binding-site interactions observed experimentally [25, 26, 28, 29].

A molecular dynamics procedure was applied to the structure of the complex of the CDK-2 or GSK-3β protein subunit with the ligand ChEMBL474807, as obtained during the docking procedure. The structure of the ligand structure was characterized using Amber force-field parameters, and the atomic charges were calculated according to the Merz–Kollman scheme via the RESP procedure [30] at the HF/6-31G* level. Each system was neutralized and immersed in a periodic TIP3P water box. The systems considered were heated to 300 K during the initial 100 ps of MD simulation, while the temperature was controlled by a Langevin thermostat [31]. Periodic boundary conditions and the SHAKE algorithm [32] were then applied for 110 ns of molecular dynamics simulation. The first 20 ns of this simulation time were used as an equilibration interval; the next 90 ns of the trajectory were used to analyze interactions between the subunits considered. The energies of the interactions between the ligand and the active site were characterized using the molecular mechanics/Poisson–Boltzmann surface area (MMPBSA) method [33]. The AMBER 11 package [34] was used in all of the molecular dynamics simulations. Structural analysis, including calculations of the hydrogen bonds present and the root-mean-square deviations (RMSDs) of atomic positions, were performed using the VMD package [35]. All of the RMSD calculations for the ligand molecule and the amino acids at the active sites were performed relative to the initial structure of the complex obtained during the docking stage. Hydrogen atoms were excluded from those calculations.

## Results

The proteins considered in this work, namely cyclin-dependent kinase 2 (CDK-2) and glycogen synthase kinase-3 (GSK-3β), exhibit high similarity in terms of the amino acid sequences within their active sites. The mechanism for inhibiting these enzymes has frequently been analyzed by computational methods. One group of compounds that exhibit high affinity for the ATP-binding pockets of the two kinases considered here includes indirubin and its analogs [22, 23, 25, 26, 28, 29]. Existing studies indicate a significant role of hydrogen bonds in stabilizing the interactions of these enzymes with ligands, with specific amino acids in each enzyme participating in the hydrogen bonds [28, 29]. For cyclin-dependent kinases, the most important roles in hydrogen-bond creation are played by the amino acid residues GUA81 and LEU83; the former participates in one hydrogen bond while the latter is involved in two [28]. A very similar situation was observed for glycogen synthase kinases, where a network of hydrogen bonds was created that involved ASP133 (one H-bond) and VAL135 (two H-bonds) [28, 29].

**Fig. 2:**
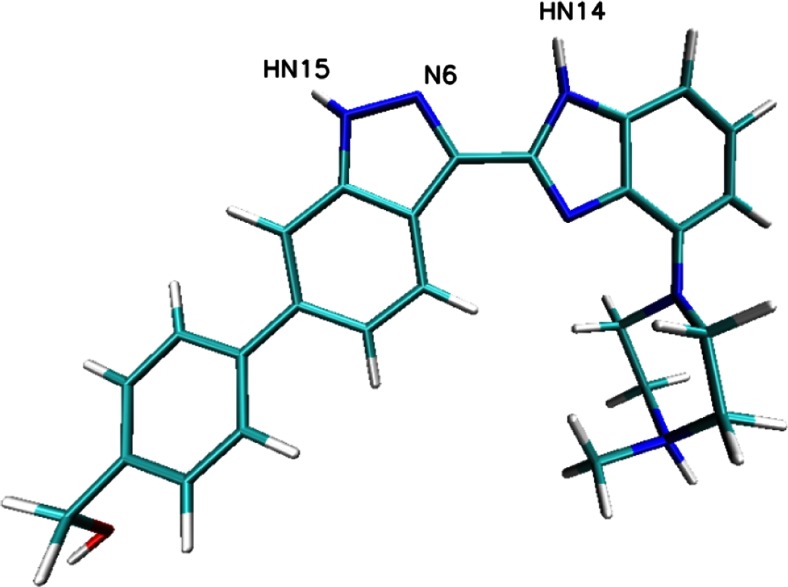
Structure of the ligand molecule ChEMBL474807; the *labeled atoms* are involved in hydrogen bonds with amino acids from the active sites of CDK-2 and GSK-3β

During the docking stage of our investigations, we used the molecule of the ligand ChEMBL474807 (Fig. 2). The nitrogen N6 and the hydrogens HN14 and HN15 were found to participate in hydrogen bonds that stabilize complexes of this ligand with kinases. After performing the docking procedure, complexes of both kinases with ChEMBL474807 were obtained. For both of the considered kinases, the ligand molecule was observed to be stabilized by three hydrogen bonds (see Fig. 1): for CDK2, these hydrogen bonds were GLU81(O)···NH14(ligand), LEU 83(HN)···N6(ligand), and LEU83(O)···HN15(ligand); for GSK 3β, the hydrogen bonds were VAL135(O)···HN15(ligand), VAL135(NH)···N6(ligand), and ASP133(O)···HN14(ligand). In both cases, the observed hydrogen bonds were consistent with existing findings for the interactions that occur in the ATP-binding pockets of the considered kinases. The binding affinities for both complexes were quite similar: −9.1 kcal/mol for CDK-2 and −10.1 kcal/mol for GSK-3β. In both cases, the standard deviations for these values did not exceed 0.1 kcal/mol.

**Fig. 3:**
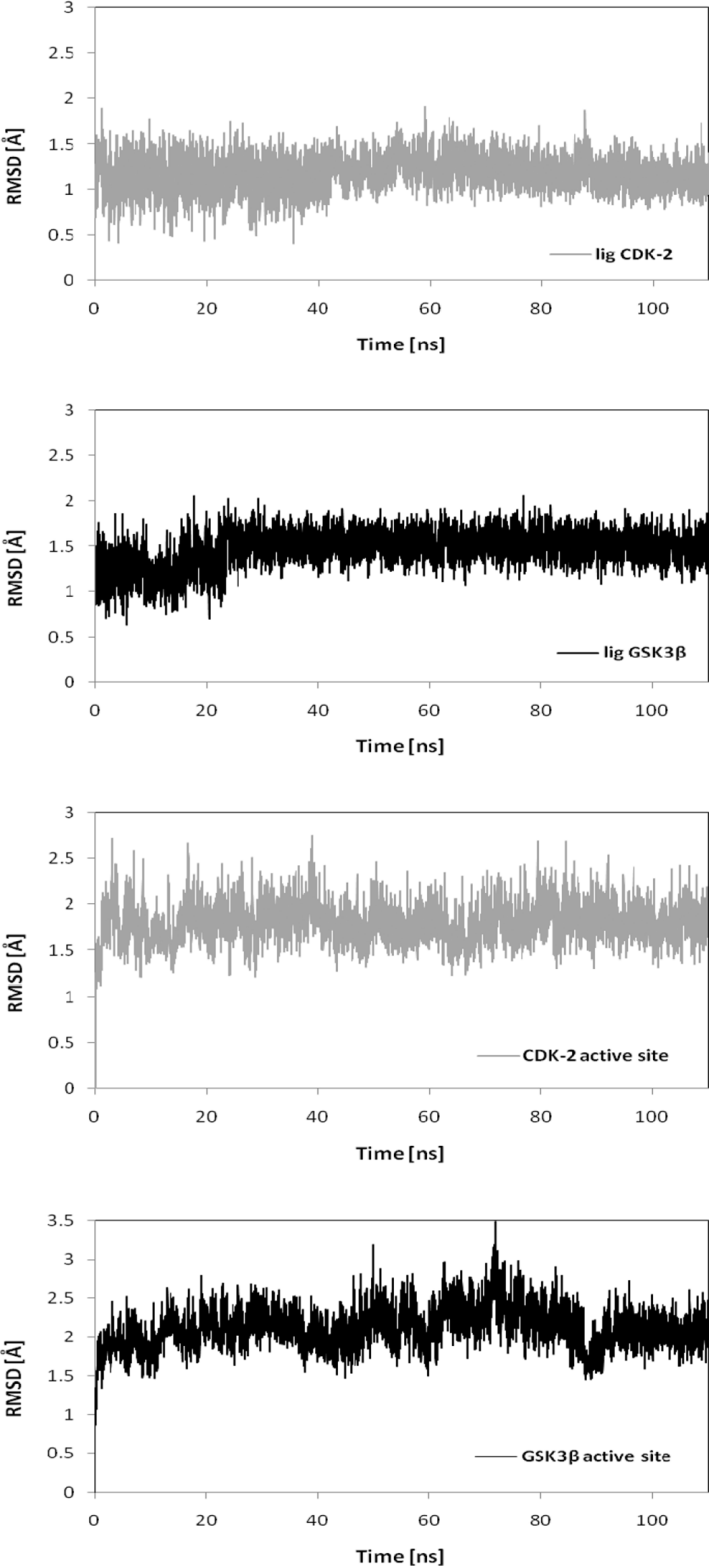
Variations in the RMSD values for the ligand and for the amino acids of the active sites of CDK-2 and GSK-3β over the course of the molecular dynamics simulation

The stabilities of the complexes obtained during the docking stage were evaluated by performing molecular dynamics simulations. These computations yielded a large group of conformations corresponding to the time evolution of ChEMBL474807 at the active site of CDK-2 or GSK-3β. The stabilities of the structures formed during the trajectories in the simulations were evaluated by calculating their mean RMSD (root-mean-square deviation) values. The graphs presented in Fig. 3 show the time evolutions of the RMSDs for the ligand molecule and for all of the amino acids comprising the ATP-binding pockets in both kinases. The RMSD values obtained led us to conclude that 20 ns of molecular dynamics simulation are sufficient to achieve equilibration. Slightly higher fluctuations in the RMSD value were seen for the GSK3B complex, an observation confirmed by the average values listed in Table 1. However, structural stabilization was seen for both complexes.

**Table 1:** Average RMSDs for the ligand and for the amino acids comprising the active site across the full molecular dynamics simulation

	Ligand when complexed with CDK-2	Active site of CDK-2	Ligand when complexed with GSK-3β	Active site of GSK-3β
RMSD	1.18	1.80	1.47	2.11
SD	0.18	0.21	0.19	0.25

*SD* standard deviation

**Fig. 4a–b:**
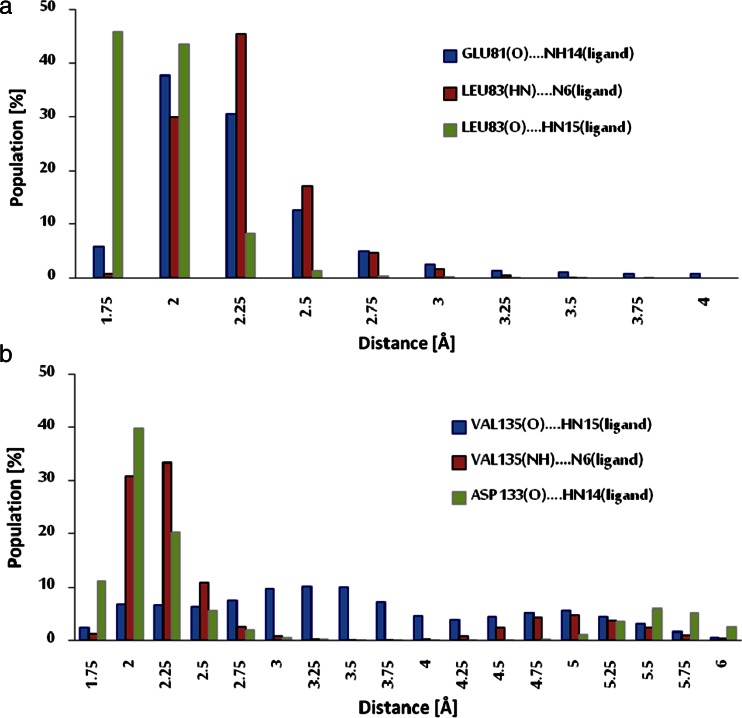
Distribution of the lengths of hydrogen bonds between ChEMBL474807 and amino acids in the active site of CDK-2 (**a**) or GSK-3β (**b**) throughout the simulation time. The hydrogen-bond lengths have been binned into 0.25-Å intervals (the length values shown represent the midpoints of the intervals)

The final 90 ns of the trajectories were used for structural analysis. The structures of both ligand–protein complexes are consolidated by various types of forces, the most important of which are hydrogen bonds and hydrophobic interactions. The results of molecular dynamics simulations confirmed the conclusions drawn from the docking results. All three hydrogen bonds between ChEMBL474807 and amino acids (GLU81 and LEU83) in CDK-2 were present throughout the simulation (Fig. 1a), although the strengths of these interactions varied over time. The strength of a hydrogen bond can be gauged from the distance between the donor and acceptor. In the ATP-binding pocket, the most stable interaction was observed to be LEU83(O)···HN15(ligand). In over 90 % of the conformations encountered during the simulation, the interaction between these atoms was a strong or moderately strong hydrogen bond (Table 2, Fig. 4). This amino acid (LEU83) also participates in the moderately strong interaction LEU83(HN)···N6(ligand), the length of which corresponded to a hydrogen bond in over 75 % of the conformations collected during the simulation. The final interaction considered was GLU81(O)···NH14(ligand). This interaction corresponded to a strong hydrogen bond in some conformations, but to a moderately strong H-bond in most conformations (70 %).

**Fig. 5a–f:**
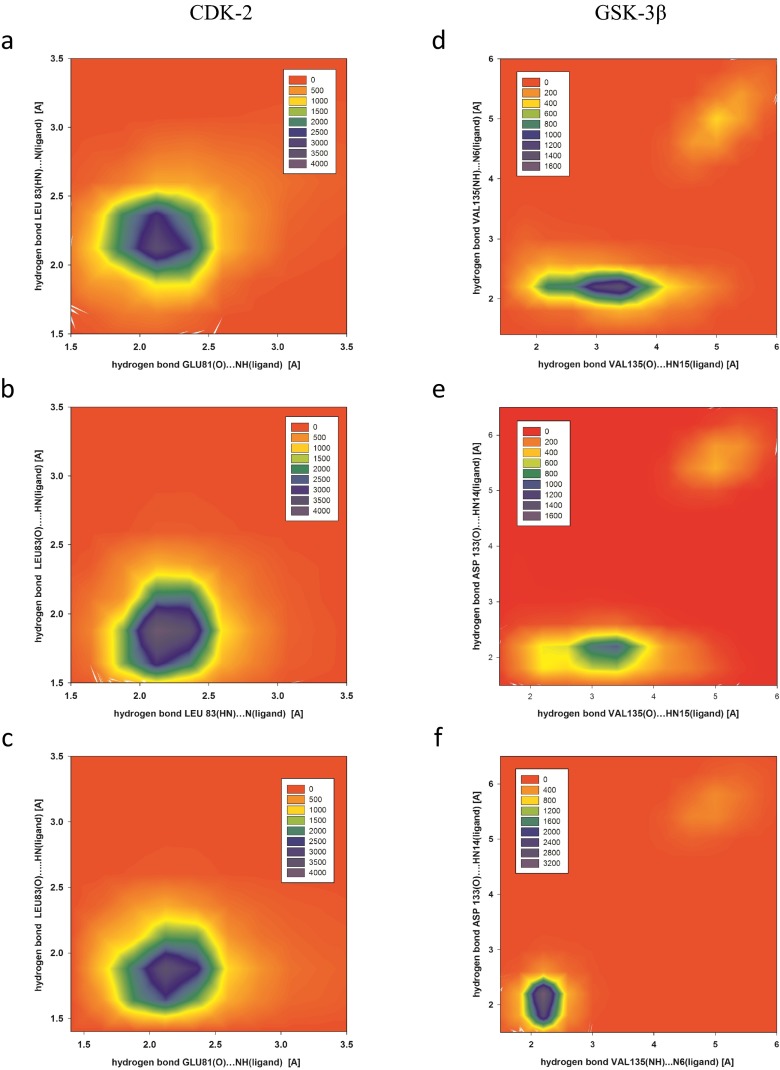
Three hydrogen bonds occurred consistently in the complex ChEMBL474807–CDK-2, according to the conformations collected during the molecular dynamics simulation of that complex, and three different H-bonds occurred consistently during the corresponding simulation of the complex ChEMBL474807–GSK-3β. This figure presents plots of one hydrogen-bond length against another for the two complexes. H-bond lengths plotted for ChEMBL474807–CDK-2: **a** GLU81(O)···NH14(ligand) versus LEU83(HN)···N6(ligand); **b** LEU83(HN)···N6(ligand) versus LEU83(O)···HN15(ligand); **c** GLU81(O)···NH14(ligand) versus LEU83(O)···HN15(ligand). H-bond lengths plotted for ChEMBL474807–GSK-3β: **d** VAL135(O)···HN15(ligand) versus VAL135(NH)···N6(ligand); **e** VAL135(O)···HN15(ligand) versus ASP133(O)···HN14(ligand); **f** VAL135(NH)···N6(ligand) versus ASP133(O)···HN14(ligand)

An important influence on the stability of each complex was the coexistence of all three of the hydrogen bonds reported above within the ATP-binding pocket, as illustrated in Fig. 5a, b, and c for the active site of CDK2: all three plots show uniform concentric distributions, which confirms that all interactions responsible for complex stabilization have uniform distribution during whole simulation without any important fluctuations indicating on conformational flexibility of analyzed system. The hydrogen-bond length data suggest that most of the conformations collected during the molecular dynamics simulations are stabilized by at least one strong and two moderately strong bonds; only a few conformations include weak hydrogen bonds. These observations confirm the high stability of the complexes obtained during the docking stage.

**Fig. 6:**
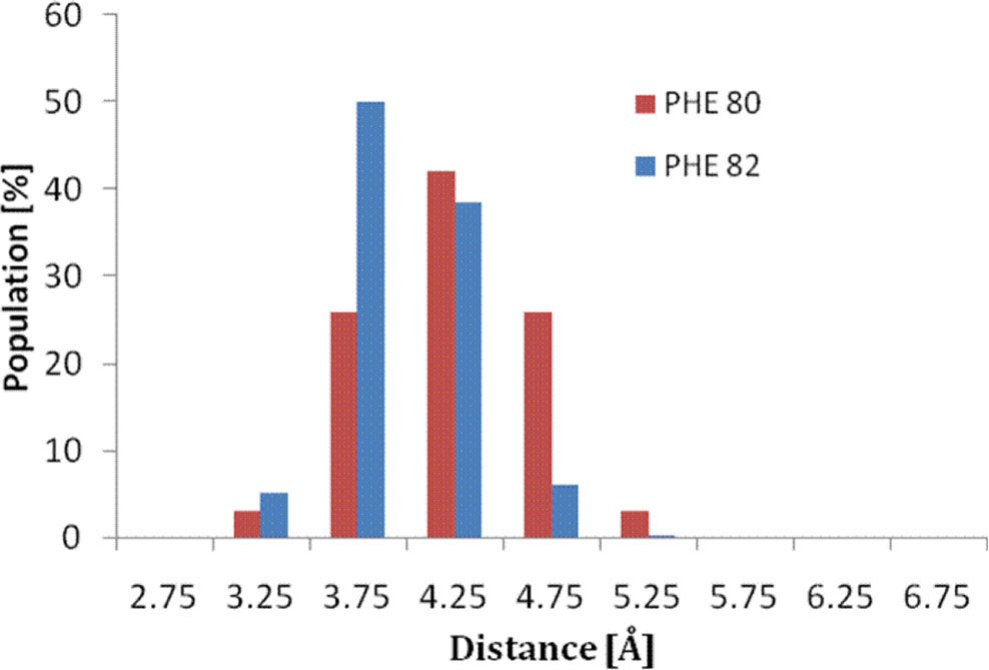
Distribution of distances between ChEMBL474807 and two phenylalanine rings at the active site of CDK-2

The next most important interactions involved in the stabilization of the ligand at the active site of CDK-2 are those between the aromatic phenylalanine rings and ChEMBL474807. The data collected (Fig. 6) indicate that, throughout the whole simulation, the distances of these aromatic rings from ChEMBL474807 and the relative orientations of the rings with respect to the ligand permit the occurrence of stabilizing interactions. During most of the simulation (PHE80: 70 %; PHE82: 85 % of the conformations), the distances between the two phenylalanine rings and the aromatic system of the ligand did not exceed 4.5 Å, leading to overlapping rings and stacking interactions in both cases.

Interactions of ChEMBL474807 with the second active site, namely that of GSK-3β, also involve three hydrogen bonds. VAL135 and ASP133 participate in these three H-bonds (Fig. 1b). The most stable hydrogen bond is ASP133(O)···HN14(ligand), which is present in more than 80 % of the conformations produced during the simulation. While this bond was usually observed to be only moderately strong, there were a non-negligible group of conformations in which this interaction was strong (Table 2 and Fig. 4). The next most important H-bond between ChEMBL474807 and GSK-3β is VAL135(NH)···N6(ligand). This hydrogen bond was found to be moderately strong in 65 % of the conformations; however, in about 20 % of the conformations, the distance between the atoms involved in this bond exceeded 3 Å, which is too long for a hydrogen bond to be effective. The third and weakest H-bond between ChEMBL474807 and GSK-3β is VAL135(O)···HN15(ligand), observed in about 40 % of the conformations collected during the molecular dynamics simulation. This H-bond was found to be either moderately strong or weak. The plots shown in Fig. 5d, e, and f confirm the previous observations made about the stabilizing interactions of ChEMBL474807 with GSK-3β. The coexistence of the two hydrogen bonds ASP133(O)···HN14(ligand) and VAL135(NH)···N6(ligand) (Fig. 5f) was found to ensure a stable complex. The position of the center of the distribution and the shape of the distribution in each plot shed light on the binding interactions considered. The shapes of the distributions in Fig. 5d and e indicate that the VAL135(O)···HN15(ligand) bond fluctuates markedly in length during the simulation. In all of the charts (Fig. 5d, e, and f), a minor fraction (∼15 %) of the conformations are characterized by interaction distances of 5–6 Å. Interestingly, the hydrogen bonds differed in their length fluctuations. In the case of VAL135(O)···HN15, unrestricted fluctuations covering the full range of bond lengths were observed, whereas stepwise changes in length were seen for the other two H-bonds (these stepped changes in bond length probably relate to changes in the conformation of the ligand ChEMBL474807 relative to the GSK-3β active site). These observations suggest the possibility of a presence of another energetically favorable conformation of ligand in the enzyme active site. Two hydrogen bonds, which formed between the ligand and oxygen atoms of two amino acids: VAL95(O)···HN14(ligand) and TYR94(OH)···N6(ligand), were observed. However analysis of the hydrogen-bond length, recorded for these conformations, suggests weak character of these interactions.

**Table 2:** Length distributions of the most common hydrogen bonds that occurred between ChEMBL474807 and selected amino acids from the active sites of CDK-2 and GSK-3β in molecular dynamics simulations

Hydrogen bond considered	Hydrogen-bond length (Å)^a^	Population (i.e., proportion of all conformations, %)
Between ChEMBL474807 and CDK-2		
GLU81(O)···HN14(ligand)	1.75	5.9
	2	37.7
	2.25	30.5
	2.5	12.6
	2.75	5.0
LEU83(HN)···N6(ligand)	2	30.0
	2.25	45.0
	2.5	17.0
	2.75	4.7
LEU83(O)···HN15(ligand)	1.75	45.8
	2	43.5
	2.25	8.4
Between ChEMBL474807 and GSK-3β		
VAL135(O)···HN15(ligand)	2	6.7
	2.25	6.5
	2.5	6.3
	2.75	7.4
	3	9.8
VAL135(NH)···N6(ligand)	2	31.0
	2.25	33.0
	2.5	11.0
ASP133(O)···HN14(ligand)	1.75	11.1
	2	40.0
	2.25	20.3
	2.5	5.7

^a^.In the table, hydrogen-bond lengths have been binned into 0.25-Å length intervals; each length listed under ‘Hydrogen-bond length’ represents the midpoint of a length interval

The energetics of both complexes during molecular dynamics simulation were analyzed. Gibbs free energy analysis was performed using the molecular mechanics/Poisson–Boltzmann surface area method. The enthalpy and entropy contributions to the Gibbs free energy were calculated for each complex.

Average energetic values for the interaction between ChEMBL474807 and CDK-2 and between ChEMBL474807 and GSK-3β are listed in Table 3. For the complex involving GSK-3β, two independent calculations were done: first, the dominant conformations of the ligand relative to the active site were characterized; second, the less common conformations were accounted for. The stabilizing interactions observed within the complex of ChEMBL474807 with CDK-2 were confirmed because their presence corresponded to the lowest value of the Gibbs free energy. The Δ*G* values for the complex including GSK-3β indicated a low affinity of the ligand for the active site, especially in the second conformation analyzed.

**Table 3:** Binding free energies (Δ*G*, kcal/mol) for the complex ChEMBL474807—CDK-2 and the complex ChEMBL474807–GSK-3β during MD simulations. Δ*H* and *T*Δ*S* refer to the enthalpic and entropic contributions to the Gibbs free energy, respectively

Energetic parameter	CDK-2		GSK-3β (1)^a^		GSK-3β (2)^a^	
	Value	SD	Value	SD	Value	SD
Δ*H*	−28.29	*4.13*	−26.01	*3.92*	−17.53	3.15
*T*Δ*S*	−10.29	*4.94*	−18.00	*7.08*	−23.73	4.89
Δ*G*	−17.68	*6.44*	−8.00	*8.09*	6.20	5.82

For the complex involving GSK-3β, two independent calculations were performed: first, the dominant conformations of the ligand relative to the active site were characterized [GSK-3β (1)]; second, the less common conformations were accounted for [GSK-3β (2)]

## Conclusions

Analysis of the properties of complexes formed by the ligand ChEMBL474807 with the kinases CDK-2 and GSK-3β revealed important differences between these complexes in their structural and energetic properties. For both complexes, conformations stabilized by hydrogen bonds (characteristic of indirubin and its analogs) were observed during the docking stage. However, the values obtained during molecular dynamics simulations indicated substantial differences between the behavior of the ligand ChEMBL474807 in the ATP-binding pocket of CDK-2 and its behavior in the ATP-binding pocket of GSK-3β; these differences were mainly in the occurrence and strength of the hydrogen bonds between the ligand and each kinase. For the complex between ChEMBL474807 and the active site of CDK-2, the greatest contribution to the ligand–kinase binding derives from the heterocyclic part of the ligand molecule, namely the atoms HN15 and N6. On the other hand, for the complex between ChEMBL474807 and the active site of GSK-3β, the heterocyclic part of the ligand molecule is much less involved in the binding process. The coexistence of all hydrogen bonds is a requirement for these complexes to remain stable. The disappearance or significant weakening of some of the H-bonds, as observed in the complex with GSK-3β, may lead to structural distortions and conformational changes. The observed differences between the complexes, which are related to differences in the frequency of occurrence and strengths of particular hydrogen bonds as well as in binding affinities, indicate that there is a higher structural and energetic affinity of the ChEMBL474807 molecule for CDK-2 than for GSK-3β.

In conclusion, the structural and energetic data presented here indicate a significant difference between the affinity of ChEMBL474807 for CDK-2 and the affinity of ChEMBL474807 for GSK-3β.
